# From Anxiety, Depression to Major Neurocognitive Disorders: Exploring Their Association With the Risk Factors for Dementia

**DOI:** 10.7759/cureus.101994

**Published:** 2026-01-21

**Authors:** Ali A Sulais, Nada Al Zamil

**Affiliations:** 1 Child and Adolescent Psychiatry, McGill University, Montréal, CAN; 2 Mental Health, King Fahad Specialist Hospital, Dammam, SAU; 3 Psychiatry, King Fahad University Hospital, Khobar, SAU

**Keywords:** dementia neurodegeneration, dementia prevention, neurocognitive disorders, psychiatric disorders neurologic diseases, psychotropic medications

## Abstract

Dementia presents an escalating public health challenge worldwide. While traditional risk factors such as metabolic syndromes and lifestyle choices have been widely examined, growing evidence highlights the role of mental health conditions in the development of dementia. Our manuscript is a narrative review of peer-reviewed systematic reviews and meta-analyses examining the association between common psychiatric disorders, namely depression, bipolar disorder, anxiety, post traumatic stress disorder (PTSD), schizophrenia, and the onset of neurocognitive decline. Additional factors such as chronic stress, sleep disturbances, and exposure to traumatic life events are also considered. Proposed mechanisms include endocrine dysregulation, neuroinflammation, and altered immune responses. The review further explores how psychopharmacological interventions may influence cognitive outcomes later in life. These insights aim to enhance clinicians’ ability to identify at-risk populations and inform preventive strategies.

## Introduction and background

The term major neurocognitive disorder (MNCD) (formerly known as dementia) is an umbrella that encompasses several disorders, including but not limited to Alzheimer’s Disease (the most common, hence the most famous), vascular dementia, Lewy body dementia, frontotemporal dementia, and others [1]. The shared feature of these diagnoses that makes them fall under the definition of MNCD is that they involve a progressive and substantial decline in cognitive functions (e.g., memory, language, visuo-spatial abilities, executive functions, etc.) that interferes with the independent functioning of the individual in the activities of daily living [2]. The neuropsychiatric disorder, MNCD, is a growing global concern with the increasing number of cases. Today, more than 55 million people live with the diagnosis around the globe. Annually, around 10 million new people are diagnosed, which is approximately equal to one new case every 3.2 seconds [3]. Due to the ongoing advances in healthcare and medicine, the number is expected to rise further, driven by longevity. According to Alzheimer’s Disease International (ADI), the number of cases is expected to double every 20 years, reaching around 139 million cases in 2050 [4]. MNCD is currently considered to be the seventh leading cause of death, and among the major causes of dependency and disability among the senior population [3].

MNCD has significant impacts on patients, their families, and the economy. In patients, emotional and physical struggles can be caused or amplified by MNCD, for example: depression, agitation, infections, poor nutrition, falls and fractures, and even death [5]. For families, caring for a patient with dementia can bring about social isolation, physical fatigue and illness, and financial challenges [6]. In 2019, MNCD cost the international economy around 1.3 trillion US dollars [3]. 

Only 15% of MNCD cases are due to reversible causes [1]. This means that no curative treatment exists until now for the vast majority of cases, and management is mainly focused on stabilizing the symptoms and slowing the disease progression [1]. This fact sheds light on the importance of preventing the disease at first place. Many classic strategies were recognized for a long time as protective against MNCD. These include enhanced physical activity, balanced healthy diet, smoking cessation, as well as controlling vascular risk factors (for example: diabetes mellitus, hypertension, dyslipidemia, etc.) [7].

However, during the 1990s, a new perspective emerged in understanding the risk of major neurocognitive disorders, centred on a key question: Is depression a risk factor for dementia or cognitive decline? This question was the title of a review article published in 2000 [8]. Given the heterogeneity of the literature and the need for an integrative, clinically oriented synthesis, a narrative review approach was adopted.

Methodology

This manuscript constitutes a narrative, integrative review. A narrative approach was chosen due to the heterogeneity of the literature across various psychiatric diagnoses and study designs, as the objective of the review was to deliver a clinically oriented synthesis. A systematic literature search was performed in PubMed and Google Scholar between March 2024 and November 2025. The review concentrated on peer-reviewed systematic reviews and meta-analyses published in English that investigated the associations between psychiatric conditions and the subsequent development of MNCD later in life. This methodological strategy was adopted to emphasize higher-level evidence and clinical significance. Primary studies, case reports, and non-peer-reviewed articles were excluded. Psychiatric conditions included were selected based on their high prevalence, clinical relevance, and their impact on patient care. Findings were synthesized narratively, with particular attention to consistency across reviews, proposed biological and psychosocial mechanisms, clinical implications, and limitations identified by the original authors.

The methodological rigor was supported by restricting inclusion to systematic reviews and meta-analyses and by interpreting findings within the context of reported heterogeneity and limitations. Articles were selected based on relevance, methodological quality, and their contribution to conceptual or clinical understanding of the topic, rather than through a predefined quantitative screening or study-count process.

## Review

MNCD and depression, the trace of stress 

“Diminished ability to think or concentrate” is a criterion for the diagnosis of major depressive disorder (MDD) as per the Diagnostic and Statistical Manual of Mental Disorders (DSM-5) [2]. On the other hand, depression is a common symptom in patients living with MNCD [5]. Earlier in 1961, LG Kiloh [9] coined the term pseudodementia to describe a condition in which severe depressive cognitive impairment can mimic dementia. This bidirectional relationship between depression and MNCD has long been established. What we know about this link can be traced to the seventies of the past century, a time when interest in stress and its ramifications was rising after the work of Hans Selye on the Stress Theory [10].

Stress can indirectly increase the risk of dementia by predisposing the individual to cardiovascular diseases, obesity, and diabetes mellitus, which are established risk factors for MNCD [11]. But is it possible that stress directly increases the risk for MNCD? Today, we know that chronic stress has a major impact on three important bodily systems, namely the central nervous system (CNS), the endocrine system and the immunological system. 

The hippocampus is a brain structure that is part of the temporal lobe. Its main functions are learning and memory. However, it is also a part of the brain's limbic system, which is responsible for the regulation of mood, pleasure, appetite, and motivation, along with other emotions and sensations [12]. This part of the brain is the primary, and usually the first atrophied part of the brain in patients with Alzheimer’s Disease [13]. One longitudinal study has revealed that chronic life stress can lead to decreased brain volume, particularly in the hippocampus [14]. 

But how does the body respond to stress? To answer this question, we move now to the next system, the endocrine. At times of stress, the body secretes the well-known hormone, adrenaline, in addition to activating a system composed of three different endocrine glands, named the Hypothalamus-Pituitary-Adrenal axis (HPA axis). Activation of the latter leads to the secretion of a cascade of hormones, ending with glucocorticoids, mainly corticosterone [15]. In case of chronic exposure to stress, the body attempts to adapt by hyperactivating the HPA axis. This hyperactivity needs to be controlled as it can lead to serious health issues, including diabetes, coronary artery disease, impaired immunity, osteoporosis, and neurotoxicity. The latest can manifest by atrophy of various brain structures, among them, the hippocampus, which is in turn involved in the negative feedback inhibition of the HPA axis by bringing down this hyperactivity [16,17].

The consequences of a hyperactive HPA axis following chronic stress do not stop here. It compromises immune system function in general and leads to a state named neuroinflammation in the brain, in which there is a high release of pro-inflammatory cytokines. This state leads to a disturbance in the normal brain cell functions, eventually causing decreased neurogenesis and impaired memory [18].

Here, we can see that the effects of stress on the three bodily systems are interrelated. In patients with depression, all these derangements are seen. A reduced hippocampal volume, HPA hyperactivity and high levels of proinflammatory cytokines [19-22]. The article mentioned earlier [8], as a question, concluded by stating, “There is sufficient evidence to take seriously the possibility that depression is a risk factor for dementia and cognitive decline.”

A systematic review and meta-analysis of 18 longitudinal studies [23] found that patients with depression had a higher risk for dementia. Currently, depression is the only mental disorder listed by the World Health Organization as an established modifiable risk factor for MNCD [3]. Another meta-analysis of 26 studies showed that patients who presented with depression, or had a history of experiencing depression, had a 1.82 times higher risk of developing dementia compared to those who didn’t show depressive symptoms at the time of the study [24].

On the mood continuum, bipolar disorder 

A systematic review published by Velosa et al. [25] concluded that individuals with bipolar disorder are at a greater risk for dementia than the general population, and interestingly, even more than individuals with MDD. The review also highlighted the apparent role lithium plays in reducing the risk of future dementia in patients with bipolar disorder. This fact is consistent with an earlier interesting finding that long-term exposure to lithium in drinking water is associated with a lower incidence of dementia [26]. However, the potential protective association of lithium should be interpreted cautiously, as observational findings may be influenced by confounding by indication and differences in clinical characteristics among treated populations.

Another systematic review published by da Silva et al. [27] reported similar findings in terms of increased risk of dementia with bipolar disorder as well as MDD. This review elaborated that the risk was greater with the higher number of depressive episodes and severity. According to this review, the risk for dementia was found to be higher in patients with bipolar disorder than in patients with MDD. This fact can be potentially explained by the usually earlier onset, higher severity, lower quality of life and poorer adjustment in patients with bipolar disorder compared to patients with MDD. 

Anxiety, what is beyond, is just a worry

Anxiety disorders are among the most common mental illnesses universally [28]. They affect men and women, the younger and the older, and people of different socioeconomic statuses. However, people usually live with their anxiety for many years before they present for treatment, if ever [1]. Multiple systematic reviews and meta-analyses reported that anxiety significantly increases the risk for MNCD [29-31]. One of the reviews suggested that treating or preventing anxiety can potentially reduce the incidence of dementia. Furthermore, another meta-analysis [30] elaborated on the risk for earlier onset of MNCD, and the particularly increased risk for Alzheimer’s and vascular dementias with anxiety. A potential mechanism highlighted in the review was the link between anxiety and vascular dementia through increasing the risk for cardiovascular disease, as anxiety can lead to vascular damage, and hence dementia, by increasing risk for hypertension, atherosclerosis, and hypercoagulability [30]. Anxiety also increases the risk for depressive disorders and substance misuse, which are additional risks for dementia as well. In summary, chronic anxiety can lead to memory impairment directly by increasing steroid levels [14], and indirectly through increasing the risk of other risk factors for MNCD, as well as leading to an inactive lifestyle and minimal social contact, which are important protective factors against MNCD [11]. 

The old dementia praecox, schizophrenia 

“Dementia praecox” was a term that Emil Kraepelin, one of the earliest pioneer psychiatrists, coined to describe what we know today as Schizophrenia [32]. The term, which means “premature dementia”, was given to these patients to denote the disturbances they had in cognition, like deficits in memory, processing, and executive functioning, which closely resembled dementia. That was until Eugen Bleuler came up with the term “schizophrenia”, a condition that might share some commonalities with dementia, but is sharply distinct in terms of dominant symptoms, course, management, and prognosis [1].

A meta-analysis concluded that schizophrenia is significantly associated with an increased risk of MNCD [33]. However, it is still not well established whether schizophrenia is a modifiable risk factor or not. Nonetheless, it is important to recognize the fact that subtle cognitive impairment is part of the natural course of schizophrenia [34]. These deficits may mimic the neuropsychological profile of patients with frontotemporal dementia, behavioral variant (e.g., deficits in memory, attention, and other executive functions, as well as personality changes). It is important not to confuse the two separate entities and to spare the diagnosis of frontotemporal dementia for those in whom there is objective evidence of the disease (e.g., brain images). 

Post-traumatic stress disorder (PTSD), how running away from memories may cause the loss of memories 

One large systematic review and meta-analysis concluded that PTSD is a strong and potentially modifiable risk factor for all-cause dementia [35]. This study is the first meta-analysis to quantify the association between PTSD and dementia risk. It included studies of PTSD in the general population, veterans, and war refugees. It revealed that in the general population, the risk of having dementia in patients with PTSD is twice the risk of dementia in those without PTSD. In veterans, those with PTSD have one and a half times higher risk of dementia than veterans without PTSD. The study proposes that the possibility of this smaller risk in veterans compared to the general population is due to the higher likelihood that this group receives treatment, which could possibly modify dementia risk. 

The exact mechanism by which PTSD increases MNCD risk is not well established yet. The above-mentioned review sheds light on the probability that PTSD and dementia may share a common underlying genetic vulnerability. Additionally, PTSD patients are particularly hypervigilant with frequent, repeated physiological and psychological arousals. This means constant or frequent activation of the body’s stress systems, with all their effects on the brain (e.g., HPA activity). Moreover, PTSD patients frequently withdraw from usual social activities due to their fear of facing reminders that can evoke memories from the traumatic event, which will make them anxious, potentially to the point of having a panic attack. This withdrawal can be significant and prolonged enough to diminish cognitive stimulation, which affects the brain’s cognitive reserve; thus, increasing the risk of MNCD [35]. 

Sleep disturbances impairing the brain’s protective mechanisms 

Insomnia is among the most common complaints in the general population. Around 10% of adults suffer from insomnia disorder, an additional 20% struggle with occasional insomnia symptoms [36]. Several meta-analyses and systematic reviews confirmed the association of sleep problems or disorders as risk factors for cognitive impairment, all-cause dementia, Alzheimer’s disease, and vascular dementia [37-39].

Several mechanisms have been proposed to explain this association. One of them is that prolonged wakefulness impairs a vital brain function named “Proteostasis”, which is maintaining a healthy balance of brain proteins. Brain proteins carry multiple crucial functions in the brain; however, if not handled and disposed of efficiently after they perform their functions, they can accumulate and set the stage for neurodegeneration to take place. Hence, their synthesis and degradation need to be balanced by proteostasis [40]. Among the proteins that can accumulate because of impaired sleep are Aβ and Tau proteins, which are neurotoxic proteins and are central in the pathology of Alzheimer’s Disease [40]. 

Impaired sleep is also found to disturb the connectivity among various brain anatomical regions, which in turn disturbs the communication among them [40]. Abnormal brain connectivity is one of the explanations of the pathology in Alzheimer’s Disease [41]. Acute and chronic sleep deficiency can also lead to neurotoxicity by inducing immunological responses, increasing levels of leukocytes and proinflammatory cytokines [42].

Stressful life events, the long-lasting effects of childhood adversities 

Above, the risk of MNCD with certain mental disorders was discussed. Going beyond that, exposure to stress itself, even if it did not result in a full clinical diagnosis, can increase the risk for MNCD. This is especially true in the case of more frequent, more severe, more chronic and earlier stressors [43]. This was found by a recently published systematic review that examined stress as a risk factor for dementia from a lifespan developmental perspective [43]. The article reported that in the case-control studies they have reviewed, people with dementia had more adverse childhood experiences than those without dementia, and chronic stressful events happening earlier in life had the greatest risk for MNCD. 

Although mixed results were found for specific stressful events, the death of a parent in childhood was found to pose the greatest risk among them. The review proposed a potential mechanism for the association between early life stress and MNCD, which is through the interference with normal brain development. This, in turn, can predispose individuals for early onset metabolic diseases, lower cognitive reserve, and poor health-related behaviors like substance use, which all can increase MNCD risk by themselves [43].

A study exploring the link between adverse childhood experiences and the risk of mental and physical illnesses suggested early detection through training teachers, social workers, and physicians as a preventive approach. It also endorsed parenting classes and training [44]. These suggestions align with Centers for Disease Control and Prevention (CDC) preventive strategies, which include enhancing economic support for families, running public awareness campaigns, and teaching children coping skills [45].

Psychiatric interventions and potential risk reduction strategies

Here, one can see how significant and remarkable the effect of mental illnesses or stress can have on late-life cognition. Going back to the initial point of this paper, which is preventing MNCD, here is a review of what the literature has to offer about the effect of various psychotropic medications on late-life cognition, which can help tailor a medication plan that takes this risk into consideration, especially in those at higher risk of cognitive disorders. 

Anticholinergic Medications

Anticholinergic drugs are occasionally used in patients with schizophrenia to manage extrapyramidal side effects that may result from antipsychotic drugs use [1]. Moreover, many of the antipsychotic and some of the antidepressant drugs have potent anticholinergic activity [46]. The cognitive impairment is one of the prominent side effects of this medication group [46]. Particularly, anticholinergic use for three or more months increases the risk of dementia by approximately 46% compared to non-use, as reported by a systematic literature review and meta-analysis by Dmochowski et al. [47]. Another systematic review and meta-analysis reported an increased risk of all-cause dementia and Alzheimer’s disease with anticholinergic agents, which takes the shape of a dose-dependent relationship [48]. A recently published Swedish case-control study looking at the use of anticholinergic medications and the incidence of dementia showed a statistically significant increase in the incidence of dementia with the use of potent anticholinergic medications. Furthermore, when examining the association with different dementia types, potent anticholinergic medications were linked more strongly to vascular and Lewy body dementia than to Alzheimer’s disease [49].

Benzodiazepines 

Benzodiazepines are used widely in psychiatry, mainly as treatments for anxiety and insomnia, in addition to other mental disorders. A number of systematic reviews and meta-analyses consistently reported that the use of this class of medications significantly increases the risk of future dementia. Risk was found to be higher with higher cumulative dose, longer duration of treatment, and when agents with longer half-lives are used [50-53].

Antipsychotic Medications

Generally, antipsychotic agents that have high anticholinergic activity, as mentioned earlier, usually have negative cognitive effects. Additionally, patients with schizophrenia tend to have subtle cognitive impairments at baseline [54]. A meta-analysis of randomized controlled trials (RCTs) compared the cognitive effects of typical antipsychotics (or first generation) to those of the atypical antipsychotics (or second generation) in schizophrenia. Results of this analysis suggest that atypical antipsychotics, compared to the typical ones, produced a slight improvement in the neuropsychological performance of patients [55]. Another meta-analysis on the long-term neurocognitive effects of antipsychotics in schizophrenia baseline reported similar findings after reviewing the RCTs that compared, head-to-head, the atypical antipsychotics to placebo or haloperidol (typical antipsychotic agent) [54]. Their results expanded to include a suggested difference between antipsychotics in their effect on overall cognition in schizophrenia patients. Quetiapine and olanzapine had the most positive scoring, followed by other atypical agents, risperidone, ziprasidone, amisulpride, and lastly, haloperidol. These cognitive benefits were particularly higher in patients who were antipsychotic-naïve and with early psychosis, which highlights the importance of early establishment of effective treatment to maximize long-term cognitive benefits, as well as all the other potential benefits from early disease control [53]. Preliminary data from a study by Kawai et al. [56] suggested that in patients with schizophrenia who took higher doses of multiple first-generation antipsychotics, dose reduction can lead to cognitive improvement. This supports the clinical recommendation of treating schizophrenia with the lowest effective dose [57].

Mood Stabilizers 

Mood stabilizers are a group of medications that are used mainly to achieve and maintain a euthymic state in patients with bipolar disorder. In this class, lithium and antiepileptic drugs are most used. A retrospective cohort study comparing lithium exposure and dementia incidence found that lithium use was linked to a reduced risk of developing major neurocognitive disorder; in both short-term (less than a year) and long-term (more than five years) users compared to non-exposed participants [58].

This result was also supported by a recent meta-analysis published by Lu et al. [59], which reported that lithium therapy seems to reduce the risk of Alzheimer’s disease, slow cognitive impairment and postpone dementia. This can be potentially explained by the well-known neuroprotective effects that lithium possesses [60]. Its chronic use increases the intracellular protein expression of the cortical and hippocampal neurons, as well as the extracellular brain-derived neurotrophic factor (BDNF) of the cortical and hippocampal neurons [60]. BDNF is the most widely spread trophic factor in the human brain, and is essential for neuronal growth, survival, and synaptic plasticity, which are important for memory and other cognitive functions [60,61]. On the other hand, a meta-analysis of observational evidence on antiseizure medications and risk of dementia reported that a higher risk of dementia was observed among bipolar patients treated with valproate, carbamazepine, and clonazepam [62]. 

Antidepressant Medications

Antidepressants have neuroprotective functions, and they were found to increase hippocampal body volume in medicated patients with MDD compared with unmedicated patients with MDD and healthy controls [19]. They also increase BDNF concentration in patients with depression [63], prevent or reverse apoptotic effects (cell death) caused by stress or glucocorticoids [64], and enhance neurogenesis and increase the number of cells in the hippocampus [65]. Thus, hypothetically, they can reduce risk directly by treating depression and anxiety and eliminating the risk they bring about, and indirectly, on a cellular level, by reversing the damaging effects stress induces on the brain. However, the clinical evidence still does not conclude whether they increase, decrease, or have no effect on late-life cognition. 

A nationwide study from Denmark included all patients diagnosed with MDD at their first visit during a 10-year study period. They found that patients who continued using older antidepressants (mainly tricyclic antidepressants) had a decreased dementia rate, while those who continued taking selective serotonin reuptake inhibitors (SSRIs), or newer non-SSRI antidepressants, did not have a decreased rate of dementia [66]. Another large population cohort study found no evidence of association between antidepressant use and cognitive decline over 10 years in an elderly population [67]. Nevertheless, two systematic reviews [23,68] and one meta-analysis [69] reported that antidepressant use in depressed patients may be associated with an increased risk of dementia or cognitive worsening later in life [23,68,69]. One study on a large population of community-dwelling women found similar results of increasing risk of cognitive impairment, especially with SSRIs and trazodone, while the use of tricyclic antidepressants was not associated with significant cognitive impairment [70].

## Conclusions

Stress, by itself, can exert devastating effects on the body. The cumulative effects of stress on various body systems eventually affect the brain, its connections, repair mechanisms, long-term stability, and future risk of cognitive disorders. Within this context, several mental illnesses-most notably depressive disorders, but also anxiety and trauma-related disorders, bipolar disorder, and psychotic disorders can be recognized as risk factors for major neurocognitive disorder, with varying strength of evidence across diagnoses. It might not be possible to prevent stressful events from happening, but it is always possible to find ways to turn around them around, stopping them from developing into full-blown mental illnesses, or having long-lasting consequences on the brain, including cognitive disorders.

Reducing stress exposure, improving problem-solving and stress management strategies, implementing appropriate psychotherapeutic interventions and the judicious use of psychopharmacotherapy; all can be protective factors considered for mitigating the impact of stress or mental illnesses as risk factors for late-life cognitive decline. It is also important to consider including this risk in patient education, especially for those who are reluctant to engage in treatment while struggling with, for example, depression, for which there is strong evidence of being a risk factor for future cognitive impairment. At the end, regardless of their association with cognitive disorders, mental illnesses indeed cause an innumerable amount of distress and interfere with one’s ability to fulfil their roles successfully. Therefore, it is essential to carefully consider the presence of mental illnesses and address them promptly and efficiently.
